# The chloroplast genome of *Prunus dielsiana* (Rosaceae)

**DOI:** 10.1080/23802359.2019.1688723

**Published:** 2019-11-13

**Authors:** Kai Zhao, Yuzhen Zhou, Yan Zheng, Bin Chen, Wei Ziling

**Affiliations:** a College of Life Science, Fujian Normal University, Fuzhou, PR China;; b College of Landscape Architecture, Fujian Agriculture and Forestry University, Fuzhou, PR China

**Keywords:** *Prunus dielsiana*, chloroplast genome, natural hybridization, consanguinity

## Abstract

Recent sequencing technology helps analyze genome-level aberrance between wild species and highly domesticated cultivars by producing high-quality genomes. Herein, we established the complete chloroplast genome of *Prunus dielsiana* to lay a foundation in the future genetic comparison and modification. The chloroplast genome was 158,005 bp in length, with a large single-copy (LSC) region of 86,012 bp and a small single-copy (SSC) region of 19,121 bp, separated by two inverted repeat (IRs) regions of 26,436 bp. A total of 124 CDSs , 37 tRNA genes, and 8 rRNA genes were found. The overall GC content was 36.70%, and GC percentages ranged from 30.20 to 42.53% throughout LSC, IRs, and SSC regions. Phylogenetic analysis showed that *P. dielsiana* displayed a kinship to *Prunus campanulata* with the subsection *Cerasus* nested inside *Prunus*. This announcement of the *P. dielsiana* chloroplast genome sequence may provide insight into the interspecific natural hybridization in *subg*. *Cerasus*.

*Prunus dielsiana* is commonly known as *Cerasus dielsiana*, one of the cherry species in the subfamily Prunoideae focke under the Rosaceae order. This species is grown in a wide range of latitudes in south China from Zhejiang to Yunnan, and adapt to the elevation gradients between 500 and 1400. Unlike other cherries, *P. dielsiana* is easy to be distinguished from related taxa, covered by Tawny nap in the tissues of twigs, leaf veins calyx, etc., besides longer calyx lobes. Due to strong resistance, *P. dielsiana* forms a wide ecological adaptability, and as reported in *Prunus* there are always natural hybridizations no matter interspecific or intraspecific, what’s more, this species usually produces variations (Katayama and Uematsu 2005). *Prunus dielsiana* is a potential parent for further selection and hybridization breeding. However, related genetic information and relationship based on it have not been well established (Zhang et al. 2018). In virtue of the sequencing technology advantages that efficiently provide longer, more accurate reads, the abundant genetic information can be accessed precisely for phylogenetic research and conservation genetics (Shirasawa et al. 2019), including assembling whole plastids. Thus, we reported a high-quality chloroplast genome (cp) of *P. dielsiana* to lay the foundation in the genetic recognition and functional analysis. Comparative methods of inner or outer species in the same genus also provided a new promising approach for species natural and artificial evolution in genus *Cerasus* (Mu et al. 2018).

Leaf samples of *P. dielsiana* were collected from the breeding farm in Fuzhou (location: 26°04′51.3′′N 119°14′19.9′′E) and preserved in Fujian Agriculture and Forestry University. Total genomic DNA was extracted by modified CTAB method to avoid the influence of high polysaccharides and phenols. The frozen samples including fresh tissues, specimens, and sequenced DNA can be found in the laboratory of Fujian Agriculture and Forestry University (Voucher specimen: YT-FJ2019-7A, FAFU, 23°32′25.19′′N 120°47′57.71′′E). PE150 pair-end library strategy was adopted and sequences were obtained by the BGI-500 platform (BGI, Wuhan, China) (Mak et al. 2017). We obtained total about 6Gb clean reads after removing adapters and low-quality reads by fastp software (Chen et al. 2018) and reads were corrected by the bfc, a standalone high-performance error-correcting tool. Then the processed data were assembled by GetOrganelle version 1.5.2 flow, in which core mapping software and assembly tool were bowtie2 and Spades version 3.13.1. Random separated reads were then assembled and extended into contigs. Fragments with low sequence coverages were removed as noises during the screening progress by using Bandage version 0.8.1 and ultimately formed a high-coverage circle chloroplast. Then, clean reads were mapped to the draf cp genome to check the assembling consistency. Detailed basic information was calculated by Bioedit. The genome was preliminarily annotated for coding genes and RNA using Bandage Prime to adjust the starting position. As a result, we established a length of 158,005 bp circle chloroplast genome of *P. dielsiana* with a total GC content of 36.70%. This cp genome typically included a length of 86,012 bp large single-copy (LSC) region and a 19,121 bp small single-copy (SSC) region, separated by two 26,436 bp inverted repeat (IRs). The four parts manifested an unbalanced GC content. LSC and SSC, 34.57% and 30.20%, are interrupted by two 42.53% GC content IRs from both sides. After assessment of the assembled plastid genome, we annotated the new cp-genome by online software GeSeq. This cp genome included 124 CDSs, 37 tRNA, and 8 conserved rRNA were found, respectively. The assembled cp genome of *P. dielsiana* and related annotation information can be detected in GenBank with an accession number of MN537436.

To determine the phylogenetic position of newly sequenced *P. dielsiana*, phylogenetic analysis was conducted along with the most representative *Prunus* species and part dicotyledon chloroplast genomes. After settled， all the cp genomes were aligned following HomBlocks pipeline (Bi et al. 2018). We reconstructed a phylogeny employing the GTR + G model and 1000 bootstrap replicates under the maximum-likelihood (ML) inference in RAxML-HPC version 8.2.10 on the CIPRES cluster. The ML tree (Figure 1) was consistent with recent phylogenetic results on *Prunus*, branch that gingko and other two species set was chosen as outgroup taxa. *Prunus dielsiana* displayed a kinship to *Prunus campanulata* with the subsection *Cerasus* nested inside *Prunus*. This appearance of the *P. dielsiana* chloroplast genome sequence seems to provide insight of the interspecific natural hybridization in *subg*. *Cerasus*. We believe the presentation of *P. dielsiana* chloroplast genome helps clarify its evolutionary status in genus *Prunus*, and provides vital genomic resources for artificial breeding and fundamental researches.

**Figure 1.**
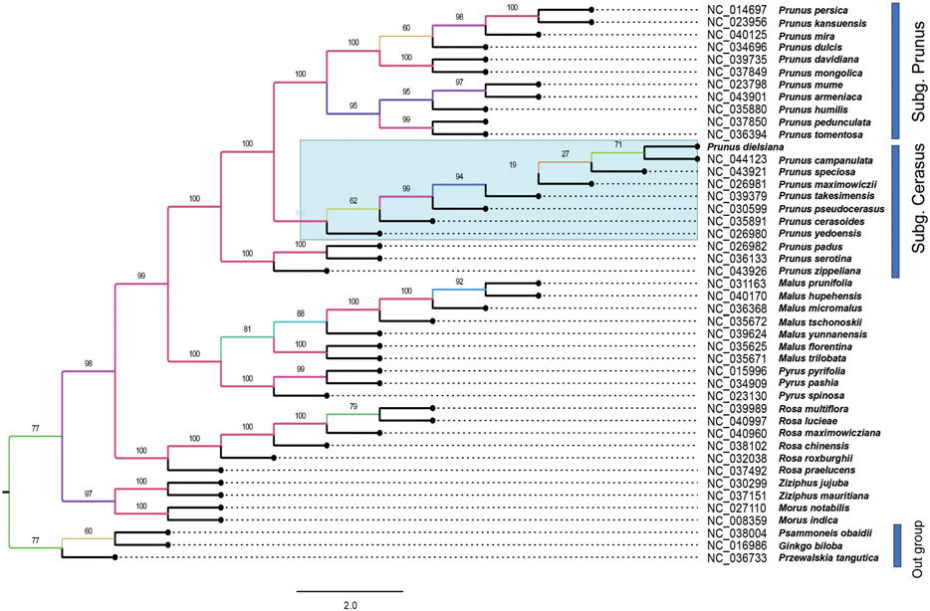
Maximum-likelihood (ML) phylogenetic tree of selected *prunus* chloroplast sequences with 1000 bootstraps. Outgroup is placed at the bottom of phylogenetic tree. Genbank accession numbers were listed before their corresponding species.
